# The role of intracellular zinc release in aging, oxidative stress, and Alzheimer’s disease

**DOI:** 10.3389/fnagi.2014.00077

**Published:** 2014-04-17

**Authors:** Meghan C. McCord, Elias Aizenman

**Affiliations:** Department of Neurobiology, University of Pittsburgh School of MedicinePittsburgh, PA, USA

**Keywords:** zinc, aging, oxidative stress, Alzheimer’s disease, apoptosis, autophagy

## Abstract

Brain aging is marked by structural, chemical, and genetic changes leading to cognitive decline and impaired neural functioning. Further, aging itself is also a risk factor for a number of neurodegenerative disorders, most notably Alzheimer’s disease (AD). Many of the pathological changes associated with aging and aging-related disorders have been attributed in part to increased and unregulated production of reactive oxygen species (ROS) in the brain. ROS are produced as a physiological byproduct of various cellular processes, and are normally detoxified by enzymes and antioxidants to help maintain neuronal homeostasis. However, cellular injury can cause excessive ROS production, triggering a state of oxidative stress that can lead to neuronal cell death. ROS and intracellular zinc are intimately related, as ROS production can lead to oxidation of proteins that normally bind the metal, thereby causing the liberation of zinc in cytoplasmic compartments. Similarly, not only can zinc impair mitochondrial function, leading to excess ROS production, but it can also activate a variety of extra-mitochondrial ROS-generating signaling cascades. As such, numerous accounts of oxidative neuronal injury by ROS-producing sources appear to also require zinc. We suggest that zinc deregulation is a common, perhaps ubiquitous component of injurious oxidative processes in neurons. This review summarizes current findings on zinc dyshomeostasis-driven signaling cascades in oxidative stress and age-related neurodegeneration, with a focus on AD, in order to highlight the critical role of the intracellular liberation of the metal during oxidative neuronal injury.

## Role of oxidative stress in aging

Brain aging is marked by gradual, general cellular dysfunction occurring as a result of structural, chemical, and genetic alterations that manifest themselves as cognitive decline, albeit with great variability among individuals. While these changes are a normal and unavoidable part of the life cycle of neurons, aging itself is also a risk factor for a number of late-life neurodegenerative disorders including Alzheimer’s disease (AD), Parkinson’s disease (PD), and amyotrophic lateral sclerosis (ALS). Although the molecular underpinnings of age-related neuropathology have not been completely resolved, one recurring explanation for the alterations observed with age, which has been revisited repeatedly for nearly 50 years, is the free radical theory of aging. This theory posits that the deleterious cellular changes that occur during aging and cognitive decline can be attributed in part to a continuous deregulation of intracellular reactive oxygen species (ROS) production over time (Harman, 1965), a phenomenon usually referred to as oxidative stress. Although this theory has been modified slightly in more recent years (Beckman and Ames, 1998), oxidative modifications caused by chronic ROS production remain recognized as a critical constituent of numerous neuropathological processes, and therefore represent a vitally important topic in the field of neurodegeneration research.

In the brain, ROS are produced as a physiological consequence of the normal oxidative processes related to cellular signaling, metabolism, and homeostasis (Lander, 1997; D’Autreaux and Toledano, 2007). Further, in addition to their presence merely as a passive byproduct of these processes, ROS also play an important and active role in a number of physiological cellular functions including gene expression, long-term potentiation, and the immune response (e.g., Sen and Packer, 1996; Knapp and Klann, 2002). As such, ROS are an integral component of a neuron’s intracellular milieu. However, while ROS are important for normal cellular processing under certain circumstances, they are more widely recognized for their deleterious role in the initiation and propagation of neuronal injury (Di Carlo et al., 2012; Lizama-Manibusan and McLaughlin, 2013). Namely, unregulated, excess production of these reactive intermediates during oxidative stress can have strong toxic effects on proteins, lipids, and nucleic acids. Moreover, ROS are also capable of triggering injurious signaling cascades that ultimately result in the demise of neurons by apoptosis or other forms of cell death (Beckman and Crow, 1993). Oxidative stress-induced cellular dysfunction can also exacerbate ROS production downstream of the initial insult, thereby maintaining the oxidative stress state in a self-propagating injury cycle that can lead to neuronal death if left unchecked (Beckman and Ames, 1998; Finkel and Holbrook, 2000).

## ROS generation and maintenance of proper oxidative homeostasis

Under normal circumstances, ROS are produced primarily as a limited byproduct of oxidative phosphorylation during the formation of ATP, which occurs via a set of redox reactions in mitochondria (Chance et al., 1979). Mitochondrial dysfunction, however, is commonly associated with neural injury cascades, and thus, in addition to their role in physiological ROS generation, mitochondria are regarded as one of principal producers of oxidative intermediates in pathological conditions. ROS themselves can also contribute to mitochondrial dysfunction either indirectly through the initiation of toxic signaling cascades that target mitochondria, or through direct damage to mitochondrial DNA (Richter et al., 1988; Esposito et al., 1999; Melov et al., 1999; Wallace, 2005). In addition to mitochondria, other intracellular generators of reactive metabolites also contribute to oxidative stress during aging, including NADPH oxidases, nitric oxide synthases (NOSs), lipoxygenases (LOXs), and peroxisomes (Halliwell and Gutteridge, 2007). Extraneuronal sources of ROS such as microglia, a non-neuronal, supporting cell involved in CNS immune responses, as well as exogenous stimuli such as UV light, ionizing radiation, and environmental toxins, also contribute to age-related neuronal dysfunction.

The brain, despite representing only 2% of the body weight, receives 15% of the cardiac blood output and accounts for 20% of the body’s total oxygen consumption (Lassen, 1959). The pronounced oxidative metabolism present in the brain results in a large generation of ROS during normal function. Neurons contain a system of enzymes and antioxidants to detoxify ROS after they are produced, as well as mechanisms to repair oxidant-induced damage once it has occurred; still, neurons become highly vulnerable to ROS-mediated damage when they are not able to adapt to ROS overproduction during times of stress (Lizama-Manibusan and McLaughlin, 2013). Therefore, oxidative stress occurs either from an overproduction of ROS, a deficiency in the antioxidant response, or both. Oxidative stress can thus be further defined as a condition in which the number of ROS produced surpasses a threshold over which they can no longer be adequately neutralized (Halliwell, 1992). Further, the deleterious consequences of oxidative stress tend to be exacerbated in the aged brain due to the combination of increased oxidant production (Gabbita et al., 1997), along with decreased ability to detoxify ROS and repair oxidatively stressed tissue (Barnett and King, 1995).

While it is not entirely surprising that cells become less able to combat the increased concentrations of injurious oxidative intermediates as they age, oxidative stress does not occur in isolation, and thus is not solely responsible for the toxic cellular processes observed during age-related neuropathology (i.e., Lu et al., 2014). Of course, many interrelated dysfunctional cellular processes coincide to lead to the immense cell loss observed during AD and other age-related neurodegenerative disorders. One such factor, which appears to be inextricably linked to oxidative stress damage in neurons, is zinc dyshomeostasis. Indeed, much like uncontrolled ROS production can have profoundly detrimental effects on neurons during aging, dyshomeostasis of intracellular zinc is also a crucial determinant of the fate of neurons in the aged brain.

## Zinc in the brain

Zinc is a ubiquitous trace element found throughout the body, including the brain, with particular abundance in the auditory brainstem, olfactory bulb, amygdala, hippocampus, and cortex (Frederickson et al., 1988, 2000; Weiss et al., 2000; Sekler et al., 2002). The cation plays a pivotal role in a multitude of cellular processes including neurotransmission, enzymatic activity, gene regulation, and structural maintenance and stabilization of proteins (Vallee and Falchuk, 1993; Choi and Koh, 1998; Frederickson et al., 2005). Due to its widespread function within neurons, intracellular zinc concentrations are tightly regulated, as proper homeostasis of the metal is critical in the maintenance of normal cellular processing. Indeed, zinc binds with high affinity to a very large number of proteins: roughly 3000 human genes, or 10% of the genome, have been identified as encoding for zinc-binding proteins (Andreini et al., 2006). While the majority (80–90%) of the zinc present in the brain is bound to metal-binding proteins, the remaining fraction is packaged within synaptic vesicles of a large sub-population of excitatory neurons (Cole et al., 1999; Frederickson et al., 2000). This synaptic or vesicular zinc is released in an activity-dependent manner, and can modulate the activation of several neurotransmitter receptors, including NMDA, AMPA, GABA_A_ and glycine receptors (for review see Smart et al., 2004; Paoletti et al., 2009; Sensi et al., 2011), as well as voltage-dependent ion channels (e.g., Grauert et al., 2014). In addition, synaptically released zinc interacts with a specific postsynaptic zinc-sensing metabotropic receptor (mZnR/GPR39) to modulate synaptic activity through its effect both on the outward chloride transporter KCC2, and on the synthesis of 2-arachidonoylglycerol, an endocannabinoid that modulates probability of presynaptic neurotransmitter release (Besser et al., 2009; Chorin et al., 2011; Saadi et al., 2012; Perez-Rosello et al., 2013).

## Zinc toxicity

It is well established that zinc exposure is toxic to neurons both *in vitro* (Yokoyama et al., 1986; Choi et al., 1988) and *in vivo* (Lees et al., 1990; Cuajungco and Lees, 1996). The overall concentration of the metal within the brain is ~150 μM, although the vast majority of intracellular zinc is normally rendered immobile through buffering by cytosolic metal-binding proteins and sequestration into organelles (Sensi et al., 2011). However, when neurons are damaged, as occurs during oxidative stress, bound intracellular zinc can be released into the cytosol, where it then triggers a number of detrimental signaling processes including those that lead to further ROS production, marking the start of a positive feedback loop involving intracellular zinc release and ROS generation (Aizenman et al., 2000; Zhang et al., 2004a). Synaptic zinc is also associated with neuronal dysfunction by its transfer from over-active presynaptic zinc-containing neurons to postsynaptic cells via calcium-permeable channels, including, but not limited to a sub-class of AMPA receptors (Weiss et al., 1993; Koh et al., 1996; Sensi et al., 2000). While proper zinc homeostasis is critical at all stages of life, the delicate balance required to keep zinc levels in check appears to be particularly precarious in the aged brain (Frazzini et al., 2006; Sensi et al., 2008; Cipriano et al., 2009; Takeda and Tamano, 2014). This is likely due to the fact that, as mentioned earlier, relative ROS levels increase as we age, and intracellular zinc fluxes appear to be very susceptible to perturbation by ROS. Indeed, zinc has been proposed as being a critical link between oxidative stress and aging (Frazzini et al., 2006). To begin to understand the mutual regulation between intracellular zinc release and ROS generation, it is first necessary to review what is currently known about how zinc is maintained within neurons and, more importantly, how the metal is liberated from metal-binding proteins during oxidative injury.

## Zinc dyshomeostasis and neuronal injury

While the largest concentration of zinc in the brain is bound to intracellular metal-binding proteins, there is a second pool, localized to synaptic vesicles of glutamatergic neurons, which constitutes 10–20% of the total concentration of the metal in neurons (Frederickson, 1989). In early studies, it had been thought that cytoplasmic influx of synaptically released zinc, referred to as “translocation”, was the primary source of toxic intracellular zinc increases during neuronal injury. This idea, however, was not consistent with later studies in mice that lacked the gene encoding ZnT3, the transporter responsible for loading zinc in synaptic vesicles (Cole et al., 1999). Despite the fact that these animals were devoid of vesicular zinc, increased intracellular concentrations of the metal and subsequent cell death still occurred, even in the apparent absence of zinc translocation from pre- to post-synaptic neurons (Lee et al., 2000). Thus, these findings strongly suggested that other sources of zinc release could also be contributing to the increased intracellular levels of the metal observed during neuronal injury. Since then, it has become increasingly clear that increased cytosolic zinc resulting from liberation from intracellular stores can be highly toxic during oxidative and other types of neuronal injury.

## Intracellular sources of zinc release

One of the main intracellular zinc binding proteins within neurons is metallothionein III (MT III). MT III is one member of a family of thiol-rich metal-binding proteins, and is the primary isoform found in neurons (Hidalgo et al., 2001). Close to a third of the 61–68 amino acids that constitute MT III are cysteine residues, cumulatively capable of binding up to seven zinc ions via their thiol side chains (Vašák and Meloni, 2011). Other MT isoforms have been shown to bind the seven zinc ions with varying affinities, supporting a role for MTs in the dynamic regulation of zinc levels dictated by the needs of the cell at any given time (Krezel and Maret, 2007). In this capacity, MT III acts as an intracellular regulator of zinc homeostasis via coordinated binding and release of the metal. Due to the very low redox potential of its thiols (−366 mV), MT III is readily oxidized, even by relatively mild oxidants. This oxidation results in the liberation of the bound zinc ions (Maret and Vallee, 1998); thus, while zinc itself is redox-inactive, its association with MT III makes it extremely susceptible to changes in cellular redox state. The release of zinc from MT III by oxidants causes a substantial increase in intracellular zinc concentration, and this single event has been established as a powerful inducer of neuronal injury (Aizenman et al., 2000).

Once zinc is liberated from MT III, it can have numerous adverse effects on neuronal function (Aras and Aizenman, 2011). As mentioned previously, mitochondria are the primary producers of ROS in neurons, and zinc plays a critical role in the regulation of mitochondrial dysfunction and ROS generation during neuronal injury. Following intracellular liberation of the cation, mitochondria have been shown to take up cytoplasmic free zinc through both the calcium uniporter as well as through an independent import mechanism that has yet to be identified (Sensi et al., 2003; Malaiyandi et al., 2005). Once sequestered in the organelle, zinc can inhibit the electron transport chain, thereby reducing mitochondrial membrane potential, which subsequently leads to an increase in ROS generation (Sensi et al., 1999; Dineley et al., 2005; Dietz et al., 2008; Medvedeva et al., 2009). Interestingly, MT III has also been shown to translocate to mitochondria and release zinc ions within the mitochondrial intramembranous space, suggesting a dynamic regulation of zinc homeostasis by the combined actions of MT III and mitochondria (Ye et al., 2001). In addition to the MT III-mediated zinc effects on mitochondria, the organelle itself has been shown to contain an independent store of zinc, which can be released during injury (Sensi et al., 2003). In that study, the authors observed that co-treatment with the thiol oxidant 2,2′-dithiodipyridine (DTDP) and the mitochondrial protonophore carbonyl cyanide 4-(trifluoromethoxy) phenylhydrazone (FCCP) results in a greater increase in cytosolic zinc than is observed by treatment with either drug alone, which the authors attribute to the existence of distinct stores of the metal that can each be liberated by unique injurious stimuli. Thus, it appears that mitochondria and MT III work in tandem to dynamically regulate intracellular availability of the cation. However, while it is known that both MT III and mitochondria can modulate zinc levels, additional work is still needed to reveal the specific contributions of each pool to the propagation of pro-death cascades following various forms of neuronal injury.

## Zinc-mediated ROS generation *in vitro*

**Figure 1:**
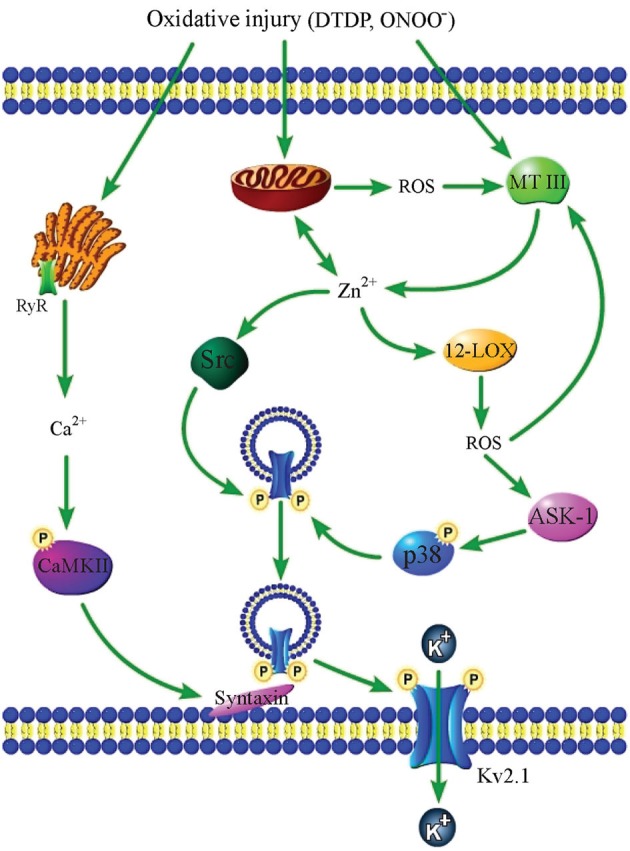
**Oxidative injury causes zinc- and calcium-dependent neuronal apoptosis**. An oxidative insult such as DTDP or ONOO^−^ exposure triggers calcium release from the ER, liberation of zinc from MT III and mitochondria, and mitochondrial ROS production. Zinc-dependent 12-LOX activation also generates ROS, which feedback on MT III to release additional zinc. Further, zinc triggers the activation of Src kinase and p38 MAPK (via upstream MAPKKK ASK-1), which then directly phosphorylate Kv2.1 channels at two amino acid residues, Y124 and S800. Calcium-activated CaMKII interacts with syntaxin to facilitate the insertion of phosphorylated Kv2.1 channels into the plasma membrane, causing the enhancement of K^+^ currents that is required for apoptosis.

In addition to triggering ROS production from mitochondria, zinc has also been found to be involved in injurious oxidant generation from a number of extra-mitochondrial sources. Interestingly, ROS generation and intracellular zinc release appear to be common constituents of a number of toxic signaling pathways in neurons. One of the better-studied zinc-mediated apoptosis cascades involves exogenous ROS-triggered zinc liberation and subsequent generation of endogenous oxidative intermediates. Specifically, cytosolic accumulation of MT III-liberated zinc can be caused by exposure to the oxidant DTDP or peroxynitrite (ONOO^−^), a physiological oxidant generated by the reaction between free radicals nitric oxide (NO) and superoxide (Beckman et al., 1990). The increase in intracellular zinc then promotes production of superoxide from the enzyme 12-lipoxygenase (12-LOX), loss of mitochondrial membrane potential, and activation of Src kinase and p38 MAPK, the latter by upstream MAP kinase kinase kinase (MAPKKK) apoptosis signal-regulating kinase 1 (ASK-1; Aizenman et al., 2000; McLaughlin et al., 2001; Zhang et al., 2004a; Aras and Aizenman, 2005). Once activated, Src and p38 directly phosphorylate the voltage-gated, delayed rectifier Kv2.1 channel at cytoplasmic residues Y124 and S800, respectively, to trigger the insertion of new channels into the plasma membrane, leading to enhanced K^+^ efflux and consequent reduction in intracellular K^+^, caspase activation, and finally, apoptotic cell death (Aizenman et al., 2000; McLaughlin et al., 2001; Redman et al., 2007, 2009). The oxidant-induced, zinc-initiated signaling cascade is accompanied by concomitant intracellular calcium release from the endoplasmic reticulum (ER), and downstream CaMKII activation, which is necessary for the exocytotic introduction of Kv2.1 channels into the plasma membrane (Figure 1; McCord and Aizenman, 2013). Exposure to NO or activated microglia-derived ONOO^−^ can also initiate this zinc- and Kv2.1-dependent apoptosis cascade (Bossy-Wetzel et al., 2004; Knoch et al., 2008), and NO exposure has also been shown to lead to cytosolic zinc accumulation in hippocampal neurons *in vivo* (Cuajungco and Lees, 1998).

Independently, NO, in combination with depletion of the antioxidant glutathione, was shown to activate 12-LOX, which results in cell death of neuronal cultures (Canals et al., 2003). While this study did not investigate the role of intracellular zinc release in this process, based on the injurious stimulus used and the downstream effects observed, these findings lend further support to the notion that various stimuli could trigger a common zinc-mediated injury cascade. Additionally, a number of studies have reported cell death following zinc-dependent activation of NADPH oxidase and nitric oxide synthase (NOS), which are the enzymes responsible for generating superoxide and NO, respectively (Halliwell and Gutteridge, 2007). While these reports focused on the effect of exogenous zinc exposure, considering the close relationship between intracellular zinc release and ONOO^−^, as well the ability of exogenously applied zinc to enter neurons, it is not unreasonable to assume that intracellular zinc stores may also play a role in NADPH oxidase and NOS co-activation (Noh and Koh, 2000; Kim and Koh, 2002). Taken together, it appears that many of the components involved in injurious mitochondrial and extra-mitochondrial ROS production, as well as the downstream processes triggered by ROS, all seem to share a common association with intracellular zinc release.

## Zinc in AD

The experimental findings summarized thus far illustrate the concept that intracellular zinc release is a common toxic event in certain forms of oxidant-induced neuronal apoptosis. As oxidative stress is a major contributor to brain aging and age-related pathology, it is feasible that zinc dyshomeostasis may also be involved in disorders associated with aging neurons (Mocchegiani et al., 2005). While the exact role of intracellular zinc in the pathophysiology of neurodegenerative disorders is not entirely clear, there is a growing body of work implicating the metal in age-related neurodegeneration. Oxidative stress-induced cell death is common between AD, PD, and ALS, as well as many other neurological disorders (Dexter et al., 1989; Olanow, 1993; Behl et al., 1994; Mecocci et al., 1994; Wiedau-Pazos et al., 1996; Smith et al., 1998). However, because the literature on the role of metals in AD is extensive, this review will focus only on zinc deregulation during AD in an attempt to paint a more cohesive picture demonstrating the fact that this metal, when unregulated, can wreak havoc on the health of neurons in an aging brain.

AD is characterized by several pathological hallmarks including amyloid plaque deposits, aggregation of neurofibrillary tangles (NFTs) composed of the protein tau in a hyperphosphorylated form, and synaptic loss and neuronal deterioration, predominantly through apoptosis (Hanger et al., 2009). Amyloid plaques are comprised primarily of β amyloid (Aβ), a ~40 amino acid long peptide generated through cleavage of the amyloid precursor protein (APP; Kang et al., 1987). Accumulation of Aβ during AD has been shown to cause neuronal apoptosis both *in vitro* and *in vivo* (Kowall et al., 1992; Loo et al., 1993). Additionally, AD and oxidative stress appear to go hand in hand, with ROS production being both a cause and consequence of Aβ aggregation (Markesbery, 1997; Butterfield et al., 2001).

## AD-induced changes in cerebral zinc

The toxic role of zinc dyshomeostasis has become an important topic in the study of AD pathology (Bush and Tanzi, 2008; Greenough et al., 2013). However, the majority of this work has focused on how synaptically released zinc contributes to AD-related neuronal dysfunction and death, and studies to characterize the effects of changes in endogenous intracellular zinc levels remain sparse. Still, the small number of reports that have examined this source of zinc support a toxic role for increased intraneuronal zinc in AD. One of the first studies to consider intracellular zinc deregulation in AD brains found that not only does the metal localize to extracellular amyloid plaque deposits, but that cytosolic zinc levels are increased as well, particularly in neurons exhibiting intracellular NFTs (Suh et al., 2000). More recently, the effect of oxidative stress on intracellular zinc mobilization was determined in neurons derived from 3xTg-AD mice, a triple transgenic AD mouse model that exhibits both Aβ and tau pathology. Importantly, this study found that intracellular zinc levels are substantially higher in 3xTg-AD neurons than in control cells following exposure to DTDP (Sensi et al., 2008). Thus, while studies of cytosolic zinc changes in AD are still in their relative infancy, these results suggest that intracellular zinc liberation could be critical for the progression of AD pathology, and that these effects appear to be mediated by the metal’s interaction with ROS.

## Zinc-mediated β amyloid aggregation and tau phosphorylation

Zinc plays an important role in Aβ aggregation, as the peptide has been shown to bind to the metal (Bush et al., 1994a). Additionally, not only does zinc exposure induce the aggregation of amyloid plaques (Bush et al., 1994b; Esler et al., 1996), but the plaques themselves are also rich in zinc, as well as copper. It is thought that Aβ is primarily responsible for inducing a state of oxidative stress during AD through direct production of oxidants (Markesbery, 1997; Huang et al., 1999; Butterfield et al., 2001), as well as through activation of microglia and subsequent generation of ONOO^−^ (Goodwin et al., 1995; Meda et al., 1995; McDonald et al., 1997; Sturchler-Pierrat et al., 1997; Weldon et al., 1998). As mentioned earlier, ONOO^−^ production originating from both neurons and microglia appears to be a key trigger of zinc-dependent neuronal apoptosis (Zhang et al., 2004a; Knoch et al., 2008). Further, hydrogen peroxide, an oxidant produced by Aβ directly (Huang et al., 1999), can cause the release of zinc from MT III, subsequently causing aggregation of Aβ. Although it was not specified if MT III is localized intra- or extracellularly, Aβ aggregates induced by endogenous zinc release are morphologically distinct from those induced by exogenous zinc application, suggesting a unique role for different zinc pools during AD (Durand et al., 2010). Interestingly, chelation of the cation facilitates the dissolution of these toxic deposits both *in vitro* (Huang et al., 1997) and in post-mortem AD brain tissue (Cherny et al., 1999). Unlike humans, aged mice and rats do not express Aβ in an aggregated form, and as such, do not exhibit the related neuropathology. Intriguingly, one notable difference between human and rodent Aβ is the peptide’s ability to bind zinc, with human Aβ exhibiting a much higher affinity for the metal (Huang et al., 2004). Thus, it is tempting to speculate that zinc could be directly responsible for the characteristic toxic aggregation of Aβ observed in AD patients, although more work is needed to definitively confirm this.

**Figure 2:**
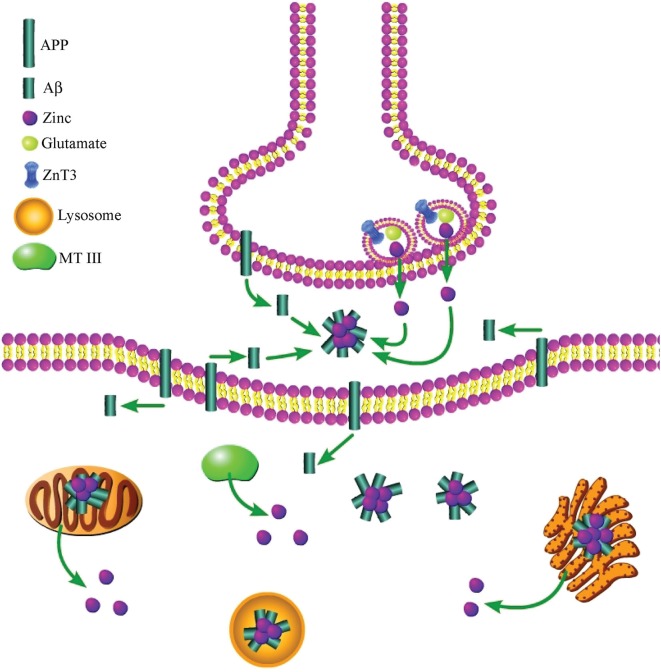
**Both extra- and intracellular zinc contribute to the toxic aggregation of Aβ during AD**. Synaptically released zinc, following secretion of zinc-containing glutamatergic vesicles, facilitates Aβ aggregation after the peptide has been cleaved from membrane-bound APP. An increase in intracellular zinc, which can be liberated from numerous sources, also enhances Aβ accumulation in the cytosol of neurons. Further, both Aβ aggregates and zinc have been found within mitochondria, lysosomes, and the ER.

While it is known that both exogenous and synaptically released zinc induce Aβ aggregation (Bush et al., 1994b; Deshpande et al., 2009), it appears that increased intracellular zinc may also play a role in this process, although conclusive work demonstrating this has yet to be undertaken. Aβ was originally identified as solely localizing extracellularly; however, other studies have shown that Aβ is also found in the cytoplasm of neurons (Turner et al., 1996; Wild-Bode et al., 1997; Gouras et al., 2000). Interestingly, a number of studies have shown that intracellular Aβ (Aβ_i_) formation precedes the appearance of extracellular Aβ, lending support to the hypothesis that an intracellular pool of the peptide is a prerequisite for extracellular plaque formation (Walsh et al., 2000; Wirths et al., 2001). However, further work is required to clarify if Aβ_i_ plays a causative role in the formation of extracellular plaques, and if intracellular zinc release promotes the induction of Aβ_i_. Nonetheless, in support of a role for cytosolic zinc, Aβ_i_ accumulation has been shown to correlate with microglial activation, increased NO production, and p38 activation (Rodrigo et al., 2004; Takuma et al., 2009), all of which are events that have previously been linked to pro-apoptotic intracellular zinc release (McLaughlin et al., 2001; Bossy-Wetzel et al., 2004; Knoch et al., 2008). Further, Aβ_i_ accumulation can be triggered by exposure to oxidants (Ohyagi et al., 2000), and its accumulation has been shown to be localized to mitochondria, which also contain zinc (Rodrigo et al., 2004), and to the ER (Hartmann et al., 1997), an organelle recently shown to be involved in zinc- and Kv2.1-dependent neuronal apoptosis (McCord and Aizenman, 2013). Zinc has been shown to be localized to the ER (Stork and Li, 2010; Taylor et al., 2012), and is released following OONO^−^ exposure (Lin et al., 2013), further bolstering a potential role for the cation in Aβ_i_ accumulation within the ER. Taken together, it is reasonable to hypothesize that intracellular zinc release could be an important factor in the accumulation of Aβ within neurons observed during AD (Figure 2).

Despite the presence of zinc in Aβ and evidence for its critical role in the aggregation of this toxic peptide, not all findings support a deleterious role for zinc in AD. In fact, exposure to low micromolar concentrations of the metal has been shown to destabilize Aβ aggregation and be protective against Aβ-induced toxicity (Garai et al., 2007). However, while low levels of zinc may protect neurons from Aβ-mediated damage, exposure to higher concentrations of the metal are toxic under otherwise equivalent conditions (Lovell et al., 1999). Like the effect of exogenous zinc on AD-related toxicity, the concentration of endogenous zinc within AD brains is also a contentious issue, although discrepancies in these studies could very well be a result of differences in the conditions under which the measurements were taken. Nonetheless, it appears that although some studies report decreased zinc levels in certain regions of AD brains (Danscher et al., 1997; Panayi et al., 2002), the overall trend supports an increase in cerebral zinc during AD (Thompson et al., 1988; Deibel et al., 1996; Danscher et al., 1997; Religa et al., 2006). Indeed, zinc chelation has proven to be neuroprotective against Aβ-mediated toxicity (Lee et al., 2004). Moreover, high concentrations of the metal have been localized to amyloid plaques and neuropil derived from AD brains (Constantinidis, 1990; Lovell et al., 1998; Suh et al., 2000), further supporting a role for increased zinc in brain areas relevant to AD pathology. Aβ-localized zinc has also been shown to contribute to AD-related damage via its effect on toxic iron accumulation (Duce et al., 2010). During AD, increases in intracellular iron can exacerbate oxidative stress and contribute to tau aggregation (Bartzokis et al., 1994; Smith et al., 1997; Yamamoto et al., 2002). Recently, APP was shown to possess ferroxidase activity that contributes to iron export and a reduction in oxidative stress in a mouse model of AD. APP ferroxidase activity is inhibited by zinc, and is negatively correlated with increased Aβ accumulation, suggesting that the zinc originated from within amyloid plaques (Duce et al., 2010). Aβ pathology can also influence the activity of certain kinases that have been closely associated with zinc deregulation and neuronal injury. Namely, numerous studies report increased phosphorylation of p38 in AD brains (Hensley et al., 1999; Zhu et al., 2000, 2001; Pei et al., 2001; Sun et al., 2003), which is also required for zinc-mediated neuronal apoptosis (McLaughlin et al., 2001). Additionally, ASK-1, the upstream MAPKKK of p38, has been linked to AD-related toxicity. Specifically, Aβ-mediated ROS production leads to activation of ASK-1 and downstream cell death in PC12 cells and cortical neurons (Kadowaki et al., 2005). Thus, many of the pathological changes that take place during AD appear to be very similar to those observed in injury related to oxidant-induced zinc liberation and downstream apoptosis.

Similar to Aβ, zinc can also directly bind to tau to facilitate aggregation of the protein into NFTs (Mo et al., 2009). Like the bimodal regulation of Aβ-induced toxicity, modulation of tau by exogenous zinc also appears to be concentration-dependent, with lower concentrations of the metal causing a decrease in phospho-tau, while higher levels cause an increase (Boom et al., 2009). Although these findings are based on exogenously applied zinc, the authors conclude that because tau accumulates intracellularly, the observed effect is likely due to translocation of exogenous zinc into the cytosol. Thus, while the effect of endogenous intracellular zinc release was not investigated, these studies nonetheless support a potential role for intracellular liberation of the cation in the regulation of tau during AD. Further support for this idea comes from evidence for accumulation of zinc predominantly within neurons that display NFTs (Suh et al., 2000). As mentioned previously, hyperphosphorylation of tau is required for its aggregation into NFTs, and many of the kinases involved in the zinc- and Kv2.1-mediated apoptosis cascade described previously also phosphorylate tau. Specifically, tau can be directly phosphorylated by both p38 (Reynolds et al., 1997) and CaMKII (Litersky et al., 1996). Further, exogenous zinc application can trigger Src kinase-dependent inactivation of PP2A, the primary phosphatase responsible for dephosphorylating tau (Liu et al., 2005, 2008). As mentioned earlier, zinc-dependent Src activity is also responsible for phosphorylating Kv2.1 channels prior to their insertion into the plasma membrane during to apoptosis (Redman et al., 2009).

## Effect of zinc on autophagic dysfunction during AD

Autophagy is a catabolic system used within cells to clear dysfunctional or unused proteins and macromolecules before they cause damage to neurons. Degradation of malfunctioning cellular components during autophagy occurs in lysosomes, which are acidic organelles containing hydrolase enzymes that facilitate the decomposition process. Autophagy is important in the clearance of protein aggregates (Johansen and Lamark, 2011), and autophagic deregulation has come to be regarded as a key occurrence in AD-related pathology (Cuervo, 2008; Nixon and Yang, 2011). It has been suggested that cell death can result from oxidative stress-induced accumulation of Aβ within lysosomes, leading to lysosomal membrane permeabilization (LMP) and subsequent release of Aβ and other toxic molecules into the cytosol (Zheng et al., 2009). In addition to toxicity caused by LMP, reduced autophagy, which has been observed in AD, can also lead to cell death due to a buildup of damaged molecules that would otherwise be degraded. In this scenario, decreased autophagy can be toxic to neurons independently of the effects of LMP. While this area of AD research is still relatively new, zinc also appears to play an important role in lysosomal dysfunction triggered by oxidative stress. Namely, oxidant exposure has been shown to cause an accumulation of zinc within lysosome-derived vesicles, as well as within the cytosol itself, leading to apoptosis of hippocampal neurons. Further, apoptosis is prevented by the zinc chelator TPEN, demonstrating that the increase in cytosolic zinc is responsible for the observed toxicity (Hwang et al., 2008).

**Figure 3:**
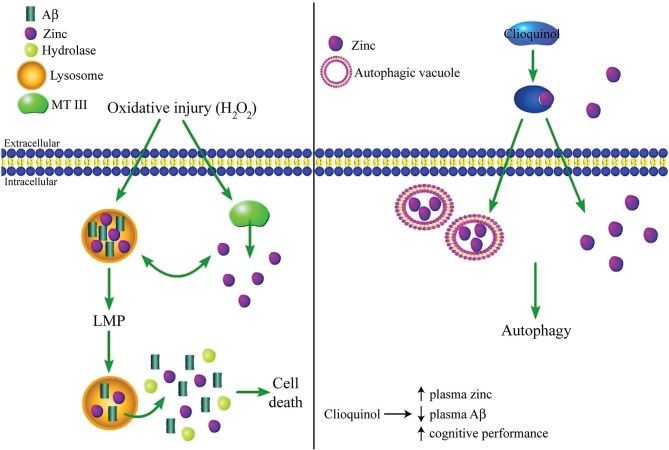
**Complex regulation of lysosomal function and autophagy by zinc. (Left panel)** Oxidative injury can lead to accumulation of zinc and Aβ within lysosomes, presumably following zinc liberation from internal stores such as MT III. This accumulation triggers LMP, which causes cell death due to the release of zinc, Aβ, and other toxic molecules like hydrolases into the cytosol. Alternatively, under other conditions zinc may have a protective effect by restoring autophagy, which is reduced in AD. **(Right panel)** Clioquinol, a clinically effective AD drug, increases intracellular zinc concentration by acting as an ionophore-like compound for the cation. Zinc accumulates in autophagic vesicles, which is necessary for the induction of autophagy and clearance of protein aggregates. However, while clioquinol has been shown to cause a reduction in Aβ and an improvement in cognitive performance in clinical trials, it can also cause zinc-mediated toxicity in otherwise healthy neurons *in vitro*, demonstrating the need for future work to clarify the role of zinc in AD-related autophagic dysfunction.

Zinc deregulation during autophagic dysfunction has also been shown to have clinical significance. Administration of the prototype AD drug clioquinol can cause a reduction of tau and Aβ, as well as an improvement in cognitive performance (Regland et al., 2001; Ritchie et al., 2003). The effect of clioquinol on Aβ aggregation was initially thought to occur because of the drug’s ability to chelate zinc (Cherny et al., 2001). However, more recent work revealed that clioquinol actually functions by acting as an ionophore-like compound to increase influx of zinc into cells from the extracellular space, which can then induce autophagy in neurons and astrocytes (Park et al., 2011). Specifically, this study found that zinc localizes to autophagic machinery (autophagic vacuoles, autolysosomes, and lysosomes), and is necessary for the clioquinol-induced clearance of accumulated huntingtin protein, which aggregates in Huntington’s disease. Thus, it appears that clioquinol may be important in degrading the Aβ and tau aggregates observed in AD, in part through its effect on extra- and intracellular zinc levels (Figure 3). However, this same study also showed that clioquinol causes zinc-dependent cell death in cortical neurons, although this experiment was not performed in the presence of Aβ or under other conditions that would mimic zinc levels or the overall cellular environment manifested in AD, and it is thus difficult to deduce the effect of clioquinol on neuronal viability during AD from this study alone.

Both abnormally increased and decreased autophagy can be detrimental to neurons depending on the circumstances in which it takes place. The studies summarized here underscore the complex nature of the role of zinc in regulating autophagic dysfunction. Indeed, it appears that changes in intracellular zinc levels can dictate if autophagy will adopt a pro-death or pro-survival function (Lee and Koh, 2010). As zinc dyshomeostasis can be observed both extracellularly and intracellularly during AD, much work remains to acquire a better understanding of the delicate balance of zinc that underlies normal cellular function, and at exactly what point metal-regulatory processes go awry to propagate the pathological effects observed during neurodegeneration.

## MT III and AD

As mentioned previously, MT III is one of the primary zinc-binding proteins within neurons, and therefore plays an integral role in maintaining homeostasis of the metal. In contrast to MT I and MT II, which are normally induced by increased levels of free metals within cells, MT III is constitutively expressed. Knockdown of MT III can lead to increased oxidant-triggered intracellular zinc levels, while overexpression of the protein substantially reduces the amount of zinc detected within neurons following oxidant exposure (Aras et al., 2009). Interestingly, numerous independent studies have reported downregulation of MT III during AD (Uchida et al., 1991; Tsuji et al., 1992; Yu et al., 2001). While these studies did not directly measure the effect of reduced MT III expression on intracellular zinc levels, they suggest that increased intracellular zinc concentration due to a reduction in MT III expression could be relevant to the pathological effects observed during the progression of the disease. Others, looking at the molecular consequences of changes in MT III during AD have shown that exogenous MT III exposure prevents the accumulation of Aβ and rescues neurons from Aβ-induced cell death (Irie and Keung, 2001), supporting a beneficial role of MT III through the sequestration of extracellular zinc. Along these lines, MT III has been suggested to be secreted by cells in the brain, albeit by not well-defined pathways (Chung and West, 2004; Manso et al., 2011). Seemingly paradoxically, a small number of studies have reported increased MT III expression in AD (Zambenedetti et al., 1998; Carrasco et al., 1999), although investigations describing this phenomenon are much less common than those reporting decreased MT III. Further, MT III can act as both an acceptor and a donor of zinc, and thus changes in MT III expression could have different effects depending on the cellular environment and redox state within neurons. Knockdown of MT III in astrocytes has also been shown to lead to a decrease in the degradative capacity of lysosomes (Lee et al., 2010), consistent with the reduced autophagy observed during AD. However, the lack of MT III also corresponds with decreased oxidant-induced zinc release, revealing the need for further studies to fully understand the role of MT III and its association with zinc in autophagic dysfunction. Nonetheless, the fact that changes in MT III expression are almost universally observed in AD suggests that deregulation of MT III is likely contributing to the zinc dyshomeostasis observed.

## Zinc and its relation to calcium deregulation during AD

Like zinc, intracellular calcium dyshomeostasis also appears to play a crucial role in AD-related pathology (LaFerla, 2002; Berridge, 2010, 2013). Simultaneous increases in intracellular zinc and calcium have been observed in a number of injurious signaling cascades related to ischemic, excitotoxic, and oxidative injury (Sensi et al., 2002; Medvedeva et al., 2009; Vander Jagt et al., 2009). However, details regarding the downstream signaling pathways activated by these two metals during oxidative injury are just beginning to arise (McCord and Aizenman, 2013). Similarly, although evidence has implicated calcium dyshomeostasis as a trigger for AD pathology (Khachaturian, 1989), if and how calcium and zinc cooperate to regulate pathological signaling during AD is still unclear. While there is some indication of a correlation between intracellular zinc and calcium levels in AD brains (Ishihara et al., 2002), direct evidence linking the two metals to pathological processes specifically associated with this disorder does not yet exist. Still, despite the lack of definitive proof, intracellular calcium and zinc release do appear to mediate many of the same processes during AD.

Just as Aβ can induce zinc liberation from internal, metal-binding stores via ROS production, the peptide can also trigger calcium release from the ER. This increase in calcium leads to ROS generation, mitochondrial dysfunction, caspase activation, and apoptosis (Ferreiro et al., 2006). As discussed earlier, all of these cellular changes have also been reported during oxidant-induced, zinc- and calcium-mediated neuronal apoptosis. Additionally, like zinc, cellular alterations induced by increased intracellular calcium contribute to intraneuronal Aβ accumulation and neurotoxicity (Pierrot et al., 2006; Demuro and Parker, 2013). Specifically, a depolarization-induced increase in cytosolic calcium can trigger phosphorylation of APP and tau, leading to subsequent Aβ_i_ accumulation and cell death (Pierrot et al., 2006). These calcium-dependent phosphorylation events are mediated by GSK-3β, a kinase known for its role in the phosphorylation of both tau and APP (Aplin et al., 1996) that has previously been shown to be activated downstream of ER calcium release (Hartigan and Johnson, 1999). Zinc can also induce GSK-3β phosphorylation, an event that corresponds to zinc-mediated activation of p38 MAPK (An et al., 2005). In another study, accumulation of Aβ_i_ was shown to trigger IP3-mediated calcium release from the ER that is necessary for Aβ_i_-induced toxicity (Demuro and Parker, 2013).

Like zinc, changes in intracellular calcium levels are extremely sensitive to oxidative stress. DTDP, a common inducer of intracellular zinc release (Aizenman et al., 2000; McLaughlin et al., 2001), has also been shown to trigger calcium release from the sarcoplasmic reticulum following oxidation of ryanodine receptors (RyRs) in cardiomyocytes (Zaidi et al., 1989). Further, DTDP-induced ER calcium release has recently been observed during zinc-dependent apoptosis in cortical neurons (McCord and Aizenman, 2013). Additionally, RyRs can also be nitrosylated by nitric oxide, leading to calcium release (Xu et al., 1998; Kakizawa et al., 2013). Deregulation of both the ER and mitochondria and the resultant effects on intraneuronal calcium levels appear to be important determinants of the progression of AD-related pathological processes (Green et al., 2008; Adam-Vizi and Starkov, 2010; Mattson, 2010). Both oxidative stress and mitochondrial dysfunction have been shown to occur early in the pathogenesis of AD (Nunomura et al., 2001; Moreira et al., 2006), and mitochondria and the ER can physically interact to regulate intracellular calcium levels in response to changes in redox state (Csordas and Hajnoczky, 2009; Hayashi et al., 2009). As mentioned previously, intracellular zinc can also regulate mitochondria and ROS production (Dineley et al., 2003; Sensi et al., 2003). Further, increased intracellular calcium triggered by glutamate exposure has been shown to contribute to mitochondrial ROS production and subsequent release of zinc from intracellular stores (Dineley et al., 2008). While these studies were not specific to any one disease, it is conceivable that similar injurious parallel processes are taking place during AD. In fact, a signaling cascade has been proposed to account for the seemingly coordinated pathways activated by zinc and calcium during AD (Corona et al., 2011). Taken together, and considering the interrelated roles of calcium and zinc in other injury models, work to reveal potential parallel processing by the metals awaits as an exciting new opportunity to enhance our understanding of the cellular signaling events underlying AD neuropathology.

## Zinc and normal brain aging

Although proper regulation of cytoplasmic zinc is crucial in determining the fate of neurons during AD and other neuropathological conditions, little is known regarding the status of intracellular release of the metal during normal, healthy aging. It is known, however, that the concentration of vesicular zinc, and the expression of ZnT3, the transporter responsible for packaging zinc into synaptic vesicles, are reduced with increasing age. Specifically, synaptic zinc levels have been shown to be decreased in the hippocampus of aged rats (Ricci et al., 1989; Amenta et al., 1990; Mocchegiani et al., 2004), and this reduction correlates with age-induced memory impairments (Guidolin et al., 1992). Further, Adlard et al. (2010) observed age-dependent memory deficits in mice lacking the gene encoding ZnT3, and also found reduced cortical ZnT3 levels in aged wild-type mice, as well as in healthy, older humans between the ages of 48–91 years. Low hippocampal ZnT3 expression and concomitant decrease in vesicular zinc concentration has also been observed in the senescence-accelerated mouse prone 10 (SAMP10) model of aging (Saito et al., 2000). While vesicular zinc levels decrease as a function of age, the total concentration of the metal in the brain appears to be unaffected by increasing age in both rodents and humans (Takahashi et al., 2001; Rahil-Khazen et al., 2002).

Though the role of cytoplasmic zinc in normal aging remains unclear, increased intracellular zinc concentration due to influx of the metal from extracellular space was recently shown to restore age-associated cognitive deficits in mice. Namely, administration of PBT2, a second-generation 8-hydroxy quinolone analog of the zinc ionophore-like AD drug clioquinol, improves cognitive ability in aged mice through a mechanism involving redistribution of zinc, resulting in an overall increase of the metal within hippocampal neurons, as well as concurrent cellular changes indicative of neurogenesis and enhanced synaptic plasticity. The status of intracellularly stored zinc was not monitored in this study, although overall brain zinc levels were unchanged by administration of PBT2 (Adlard et al., 2014). In addition to diminishing cognitive decline during normal aging, PBT2 also improves cognitive performance in mouse models of AD (Adlard et al., 2008). Thus, it seems that age-related cognitive impairment could occur through similar mechanisms under both physiological and pathological circumstances, and that cerebral zinc dynamics play an important part of the cellular processes underlying these cognitive changes. As such, determining how zinc localized to intracellular stores contributes to the cognitive deficits that accompany aging could greatly enhance our understanding of the function of the metal during normal aging.

Dietary zinc deficiency is common among the elderly, and has been attributed to dysfunction in the body’s immune response, including changes in the antioxidant defense system, which manifest in part as an increase in the incidence of infection and inflammatory processes (Kelly et al., 1996; Mocchegiani et al., 2000, 2008; Haase and Rink, 2009; Wong et al., 2013). Unfortunately, although zinc deficiency is frequently observed in the aged population, results regarding the effect of zinc supplementation on healthy aging have been somewhat inconsistent, and a consensus has yet to be reached on the benefits of such treatment (Mocchegiani et al., 2008). Most studies on the effect of zinc deficiency on aging have been focused on areas of the body outside of the brain, and thus, at present, the effects of dietary zinc deficiency on brain aging are not well understood. While it has been shown that activities of many proteins known to be modulated by zinc are altered with increasing age, there is a dearth of reports explicitly studying if and how dietary zinc deficiency contributes to these changes (for review see Mocchegiani et al., 2005). As such, additional research is needed to clarify the role of dietary zinc in the aging brain.

## Kv2.1-mediated K^+^ efflux and AD

A necessary downstream event in zinc-mediated apoptosis is enhanced K^+^ efflux through the delayed-rectifier Kv2.1 channel. Low intracellular K^+^ is a requisite step in many apoptosis pathways, as it facilitates protease and nuclease activation, cytochrome c release from mitochondria, and apoptosis-related cellular volume decrease (Bortner et al., 1997; Hughes et al., 1997; Yu et al., 1997; Maeno et al., 2000; Cain et al., 2001). Intracellular zinc release and downstream Kv2.1-mediated K^+^ efflux can both be triggered by exposure to DTDP, NO, and activated microglia (Pal et al., 2003; Bossy-Wetzel et al., 2004; Knoch et al., 2008). Further, oxidant-induced K^+^ current enhancement is prevented by the zinc chelator TPEN, demonstrating the dependence of apoptotic Kv2.1 activity on zinc (McLaughlin et al., 2001). In addition to its role in zinc-mediated apoptosis, Kv2.1 also appears to be involved in the toxic cellular processes related to AD. For a number of years, studies have demonstrated an enhancement of voltage-gated, delayed rectifier K^+^ currents following exposure of neurons and astrocytes to Aβ (Jalonen et al., 1997; Colom et al., 1998; Yu et al., 1998). More recently, though, changes specifically within the Kv2.1 channel have been identified in animal models of AD. Namely, upregulation of Kv2.1 mRNA and protein has been reported in rats injected with Aβ; this change in Kv2.1 expression is accompanied by impaired performance on spatial memory tasks (Pan et al., 2004). It is well known that deficits in acetylcholine are intimately related to AD-associated cognitive decline, thus forming the basis of acetylcholinesterase (AChE)-based therapy in the disease (Bartus et al., 1982; Francis et al., 1999). Interestingly, the AChE inhibitor galantamine, used in the treatment of AD, has been shown to cause a reduction in basal delayed rectifier K^+^ currents in hippocampal neurons (Pan et al., 2003; Vicente et al., 2010), likely arising from Kv2.1 channels (Zhang et al., 2004b).

Hydrogen peroxide exposure has been shown to directly oxidize Kv2.1 channels, leading to channel oligomerization and downstream apoptosis. Importantly, an oxidation-resistant Kv2.1 channel cysteine mutant (C73A) that prevents oligomerization also attenuates toxicity induced by Aβ exposure (Cotella et al., 2012). Interestingly, enhanced oligomerization of Kv2.1 is also observed in a mouse model of AD, although how this change in Kv2.1 structure contributes to AD-related cognition decline was not determined (Cotella et al., 2012). In contrast to reports of enhanced Kv2.1-mediated K^+^ currents during apoptosis, this study found that oxidant exposure actually decreases K^+^ currents, and the Kv2.1C73A mutation blocks this effect. However, this discrepancy could be explained by the fact that it takes ~3 h to observe the Kv2.1-mediated K^+^ current enhancement after oxidant exposure (McLaughlin et al., 2001), and this group only examined currents immediately following the oxidative insult. Further, the toxic effects of Kv2.1 channel oligomerization may occur independently of the change in K^+^ currents, as a second oligomerization-impaired channel mutant (C73S) was found to be non-conducting, yet still rescues cells from oxidant-induced apoptosis (Cotella et al., 2012). Thus, it is not yet clear how alterations in Kv2.1-mediated K^+^ currents may influence Aβ-mediated toxicity.

## Conclusions

Oxidative stress can generally be considered both a cause and an effect of the neuropathological changes seen in AD and other age-related neurodegenerative disorders. In addition, links between increased intracellular zinc, oxidative stress, and age-related neurodegeneration have been established in numerous studies, and zinc dyshomeostasis appears to be a common constituent of a multitude of pathological neuronal processes. Nonetheless, a direct causative role of intracellular zinc release in human neurodegenerative disorders has yet to be firmly established. It is known that zinc homeostasis is critical for proper brain function, and even minor disturbances to this delicate balance can trigger an accumulation of zinc, which can have extremely adverse effects on the fate of neurons in AD and related disorders, in part through an apoptotic enhancement of Kv2.1-mediated K^+^ currents. As such, modulation of intracellular zinc levels could be a particularly important target in order to protect against AD-related injurious cellular processes. However, while chelation of zinc may be an effective neuroprotective strategy *in vitro*, the potential therapeutic benefits of zinc chelation become much more complex when studying changes in zinc levels *in vivo*, in particular as zinc can also act as a neuromodulator or neurotransmitter, and it has a wide range of additional essential functions in neurons, as well as throughout the organism, including, but not limited to regulation of gene expression. This matter is thus complicated by the fact that both increased and decreased intracellular zinc can be neurotoxic, presumably depending on specific cellular conditions. Still, the fact that drugs like the zinc ionophore-like compound clioquinol have been effective in abrogating some of the pathological consequences of AD in preliminary clinical trials (Regland et al., 2001; Ritchie et al., 2003), presumably through its effect on modulating zinc levels in order to restore autophagy, indicates that the metal plays a pivotal role in the progression of AD, and that strategies targeting zinc could hold the key to finding better treatments for this currently incurable disease. Still, future work is required to determine exactly how clioquinol and its association with zinc improve the outcome of AD progression. Further, investigation into the potential off-target effects of this drug is crucial, as increased intracellular zinc, as illustrated throughout this review, is lethal to neurons in a variety of settings.

## Conflict of interest statement

The authors declare that the research was conducted in the absence of any commercial or financial relationships that could be construed as a potential conflict of interest.
